# Modulation of autophagy as a therapeutic strategy for *Toxoplasma gondii* infection

**DOI:** 10.3389/fcimb.2022.902428

**Published:** 2022-08-24

**Authors:** Ao Cheng, Huanan Zhang, Baike Chen, Shengyao Zheng, Hongyi Wang, Yijia Shi, Siyao You, Ming Li, Liping Jiang

**Affiliations:** ^1^ Department of Parasitology, Xiangya School of Medicine, Central South University, Changsha, China; ^2^ Department of Immunology, Xiangya School of Medicine, Central South University, Changsha, China; ^3^ China-Africa Research Center of Infectious Diseases, Xiangya School of Medicine, Central South University, Changsha, China

**Keywords:** autophagy, *Toxoplasma gondii*, CD40, IFN-γ, AMPK, mTOR

## Abstract

*Toxoplasma gondii* infection is a severe health threat that endangers billions of people worldwide. *T. gondii* utilizes the host cell membrane to form a parasitophorous vacuole (PV), thereby fully isolating itself from the host cell cytoplasm and making intracellular clearance difficult. PV can be targeted and destroyed by autophagy. Autophagic targeting results in *T. gondii* killing *via* the fusion of autophagosomes and lysosomes. However, *T. gondii* has developed many strategies to suppress autophagic targeting. Accordingly, the interplay between host cell autophagy and *T. gondii* is an emerging area with important practical implications. By promoting the canonical autophagy pathway or attenuating the suppression of autophagic targeting, autophagy can be effectively utilized in the development of novel therapeutic strategies against *T gondii*. Here, we have illustrated the complex interplay between host cell mediated autophagy and *T. gondii*. Different strategies to promote autophagy in order to target the parasite have been elucidated. Besides, we have analyzed some potential new drug molecules from the DrugBank database using bioinformatics tools, which can modulate autophagy. Various challenges and opportunities focusing autophagy mediated *T. gondii* clearance have been discussed, which will provide new insights for the development of novel drugs against the parasite.

## 1. Introduction

*Toxoplasma gondii* (*T. gondii*), an intracellular protozoan parasite infectious to warm-blooded animals, is estimated to infect billions of people worldwide. As the causal pathogen of toxoplasmosis, *T. gondii* can lead to encephalitis and ocular diseases and may cause severe systemic infections in immunocompromised individuals, such as patients with cancer and AIDS (Halonen and Weiss, 2013). Owing to the high prevalence of *T. gondii* infection and the difficulty of specific elimination, the identification of suitable drugs is an urgent research goal. *T. gondii* resides and proliferates in the nucleated cells of the host. Structures like micronemes and rhoptries enable the parasites to bind to the host cell membrane, invade, and form the parasitophorous vacuole (PV), a niche encapsulating the parasite and separating from host cell cytoplasm by parasitophorous vacuole membrane (PVM). The Th1 type cellular immune response mediated by interferon-γ (IFN-γ) and interleukin-12 (IL-12) is the primary immune response against *T. gondii* (Suzuki et al., 1988; Sher et al., 2003), in which dendritic cells, CD8^+^ T cells, monocytes, and natural killer cells function as the major effectors in the different stages of *T. gondii* infection (Parker et al., 1991; Dunay et al., 2010; López-Yglesias et al., 2021). Immunological mechanisms to eliminate intracellular *T. gondii* include phagocytosis, cytotoxicity, the release of nitric oxide (NO) and cytokine-dependent pathways. Among these, autophagy induced by *T. gondii* has drawn substantial attention because of its parasiticidal effects.

**Figure 1:**
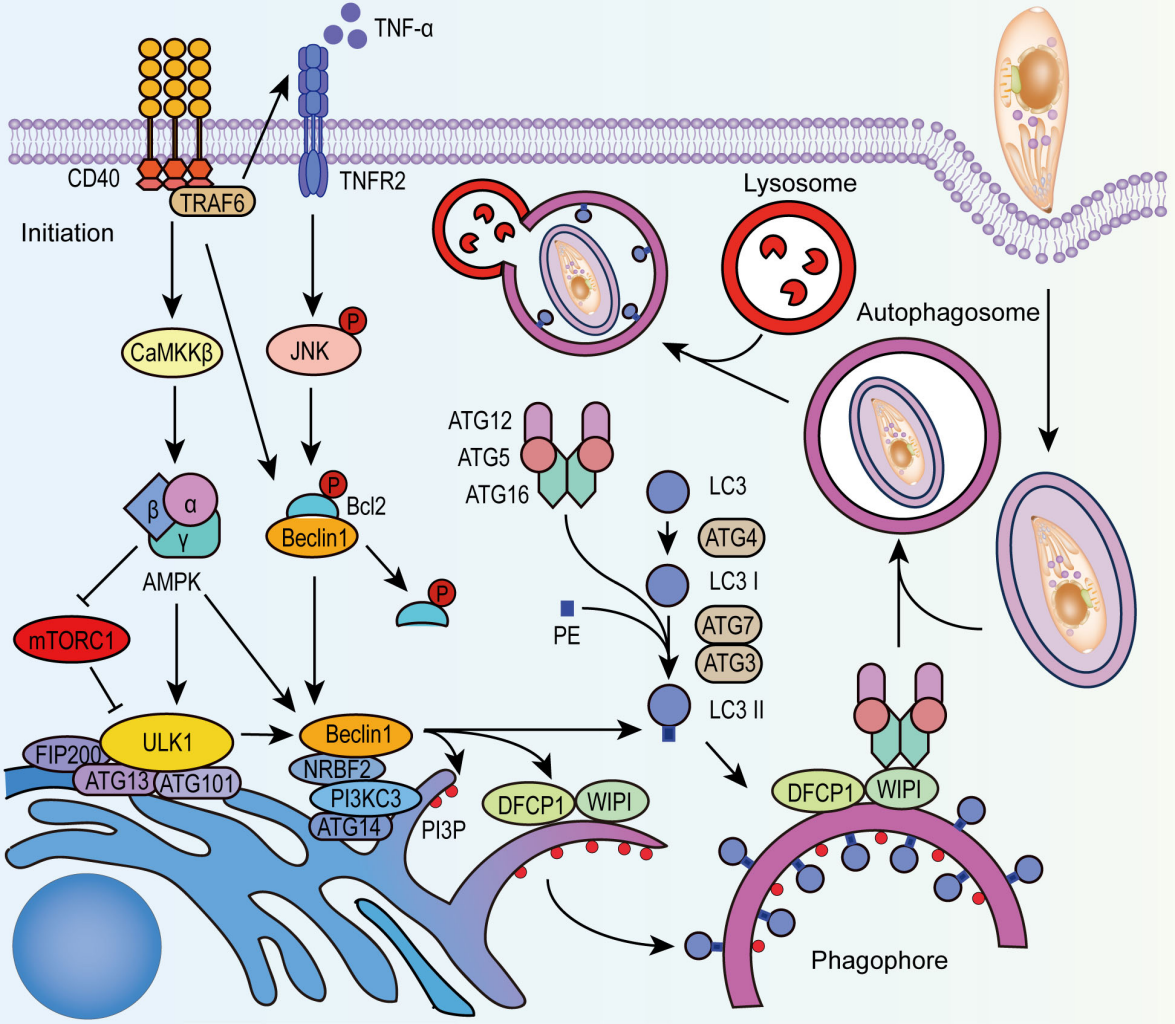
CD40 stimulates autophagy in T. gondii infection. After the invasion of T. gondii, CD40 activates ULK1 through CaMKKβ-AMPK signaling and releases Beclin1 from phosphorylated Bcl-2. AMPK also alleviates the negative regulation of mTORC1 to ULK1. CD40 promotes the activation of TNFR-JNK signaling through phosphorylation. Activated ULK1 complex and Beclin 1-PI3KC3 complex recruit PI3P, DFCP1, and WIPI to form phagophore. With the recruitment of LC3-II that is lipidated from LC3 by ATG4, ATG7, ATG3, and ATG12-ATG5-ATG16 complex, the phagophore expands and closes to form autophagosome that encapsulates PV of T. gondii, and fuses with lysosomes to degrade the parasites. PI3P, phosphatidylinositol-3-phosphate; NRBF2, nuclear receptor binding factor 2; AMBRA1, BECN1-regulated autophagy protein 1; DFCP1, double FYVE domain-containing protein 1; WIPI, WD-repeat protein interacting with phosphoinositides.

Autophagy is a highly conserved process for the maintenance of cellular homeostasis and the elimination of damaged or harmful components in eukaryotic cells. As a self-digestive lysosomal degradation system, autophagy is also regarded as a part of the innate immune response defending against intracellular microbes, such as viruses, bacteria, and protozoa (Deretic et al., 2013). There are generally three types of autophagy: autophagosome-mediated macro-autophagy, micro-autophagy, and chaperone-mediated autophagy. Macro-autophagy inhibits *T. gondii* infection depending on the formation of a parasite-containing autophagosome and its fusion with lysosomes (**Figure 1**). The basic steps of macro-autophagy include (I) initiation: in which the assembly of the Unc-51-like kinase 1 (ULK1) complex initiates the pre-autophagosome structure formation; (II) nucleation-elongation-maturation: in which phosphatidylinositol 3-kinase catalytic subunit type 3 (PI3KC3) interacts with ULK1 to form phagophores, with the coupling of phosphatidylethanolamine (PE) and microtubule-associated protein light chain 3(LC3) facilitated by the autophagy protein (ATG) complex (ATG12-ATG5-ATG16), the phagophore membrane elongates and closes to form mature autophagosome; (III) fusion of the mature autophagosome and lysosome; (IV) degradation of engulfed contents for recycling uses or discarding (Cao et al., 2021). It is reported that CD40 is the key initiator of canonical autophagy against *T. gondii* infection (Andrade et al., 2006; Van Grol et al., 2013) in both hematopoietic cells (such as macrophages) and non-hematopoietic cells (such as fibroblasts and epithelial cells). Signaling molecules such as mammalian target of rapamycin (mTOR) and 5′ AMP-activated protein kinase (AMPK) function as important regulators during this process (Ling et al., 2006; Liu et al., 2016). Besides, IFN-γ mediated signaling pathways and the oxidative stress in host cells have synergistic effects with autophagy to eliminate *T. gondii* (Ohshima et al., 2014).

To avoid autophagic killing from the host, *T. gondii* has evolved strategies to manipulate host autophagy signalings. By excluding host proteins and inserting its own to the PVM, the extensively modified PV not only allows the transport of nutrients, but also protects *T. gondii* from host targeting and degradation (Lee et al., 2013). Meanwhile, rhoptry proteins (ROP), such as ROP5, ROP16 and ROP18, are released from the apical structure rhoptry during the invasion and located on the cytosolic side of the PVM, which inhibit the accumulation of host immunity related GTPases (IRGs) on PVM to protect PVM integrity (Hermanns et al., 2016). ROP16, released and translocated into the nucleus, affects host gene expression by intersecting host STAT pathway (Saeij et al., 2007). However, there is little evidence indicating ROPs could directly regulate autophagy, and the correlation between various ROPs and autophagy pathways needs further exploration. By phosphorylating the epidermal growth factor receptor (EGFR), *T. gondii* could alter autophagic targeting and suppress autophagy-mediated clearance (Muniz-Feliciano et al., 2013; Portillo et al., 2017). *T. gondii* could also exploit the endo-lysosomal system and recruit autophagosomes to provide nutrition, especially fatty acids, for its proliferation. Such interactions between *T. gondii and* host autophagy can be targeted for future drug development. A clear understanding of the parasiticidal mechanisms mediated by CD40 and IFN-γ and the interplay between host cells and *T. gondii* is expected to provide a basis for infection control. In this review, we present the mechanisms of autophagy during *T. gondii* infection. Targets to enhance host cell autophagy or prevent *T. gondii* from blocking autophagy process with potential therapeutic value are highlighted. With bioinformatics tools, candidate drugs targeting CD40/IFN-γ-related pathways for anti-toxoplasmosis therapy are also summarized from DrugBank database, which are a subject for further exploration.

## 2. *T. gondii* infection and current treatments

Being firstly identified in 1908, *T. gondii* is estimated to infect about one-third of the world’s population (Flegr et al., 2014). *T. gondii* is a zoonotic protozoan parasite belonging to genus *Toxoplasma* of Phylum Apicomplexa (Khan et al., 2005). In early studies, 106 *T. gondii* strains isolated from North America and Europe were initially classified into three classical clonal lineages, type I, II, and III, based on six marker genes (Howe et al., 1997). Further analyses using PCR-RFLP (polymerase chain reaction-restricted fragment length polymorphism) based on 10 marker genes have identified hundreds of atypical genotypes with differences in virulence and prevalence reported in *Toxoplasma* genome database. Type I shows greater lethality in mice and is associated with acute outbreaks. Type II is the dominant strain in Europe and North America and the most common strain isolated from clinical cases of toxoplasmosis (Howe et al., 1997).

Most patients infected by *T. gondii* are asymptomatic or present mild symptoms, but some are as severe as lethal toxoplasmosis, depending on host immune response (Montoya and Liesenfeld, 2004). In immunocompromised hosts, such as patients with AIDS/HIV, the newly acquired or the reactivated chronic infection can lead to severe encephalitis, chorioretinitis, or myocarditis (Rodgers and Harris, 1996; Eza and Lucas, 2006). Primary infection of *T. gondii* during pregnancy may result in abortion and congenital toxoplasmosis. It is noteworthy that *T. gondii* infection is related to psychiatric disorders in some subclinical cases, as numerous studies have shown the correlation between schizophrenia and *T. gondii* infection (Xiao et al., 2018). Chronic toxoplasmosis is an significant risk factor for schizophrenia, which might be attributed to the dysregulation of neuroendocrine and the neurotransmitter imbalance caused by chronic neuroinflammation, while psychiatric disorders also increase the exposure risk of *T. gondii* (Yin et al., 2022).

Since Sabin and Warren (1942) discovered the anti-toxoplasmosis effect of sulfonamides as the antagonist of folic acid, Eyles and Coleman subsequently reported the synergistic effects of sulfonamides and pyrimethamine, which have become the standard therapy for toxoplasmosis (Dubey, 2008). However, increasing drug resistance and serious adverse reactions of sulfonamides were reported (Shammaa et al., 2021), leading to an urgent need to develop new treatment methods. Spiramycin is used to reduce vertical transmission in pregnant women due to its low toxicity and impermeability to the blood-placenta barrier (Foulon et al., 1999). Atovaquone showed therapeutic potential in the salvage therapy of *Toxoplasma* encephalitis or in patients who could not tolerate the sulfonamides treatment (Torres et al., 1997). Clindamycin, clarithromycin, and azithromycin also serve as alternatives to sulfonamides therapy, but the efficacy of these alternative antibiotics remains controversial (Dunay et al., 2018). Furthermore, several vaccines have been reported to increase the immune response and survival rate in mice (Chu and Quan, 2021). However, substantial work is needed for clinical translation. To reduce the adverse reactions and drug resistance of anti-*T. gondii* therapies, it is essential to further explore the anti-*T. gondii* mechanisms and identify novel targets for *T. gondii* treatment.

## 3. The interplay of *T. gondii* and host cell autophagy

Autophagy is an important cellular mechanism to eliminate *T. gondii* and can be mediated by different signaling pathways. In *T. gondii*-infected host cells, autophagy is mainly stimulated by CD40. In addition, IFN-γ can regulate the growth or elimination of *T. gondii* by inducing an ATG-dependent signaling cascade (Sasai et al., 2018).

### 3.1 CD40 stimulates autophagy pathways in host cells infected by *T. gondii*

CD40 is a member of the tumor necrosis factor receptor (TNFR) superfamily that is expressed on both antigen-presenting cells and various non-hematopoietic cells. It contributes to cell-mediated immunity against *T. gondii* following the interaction with the ligand CD154 (Reichmann et al., 2000). In 2006, Andrade et al. found that CD40 could activate macrophages and alter the nonfusogenic nature of PVs, thereby inducing the autophagic degradation of *T. gondii* both in human and mouse cells (Andrade et al., 2006).

CD40 regulates autophagy in the infected cells *via* several pathways (**Figure 1**): (I)CD40 activates AMPK depending on Calcium/calmodulin-depend kinase kinase-β (CaMKKβ) resulting in the phosphorylation of ULK1 (Liu et al., 2016), and this process can be inhibited by mTOR complex1 (mTORC1). (II) CD40 stimulates the production of TNF-α, activates the MAPK/JNK signaling pathway and phosphorylates Bcl-2, contributing to the dissociation of Bcl-2 from Beclin 1-Bcl-2 complex (Liu et al., 2016). Similarly, CD40 downregulates the Beclin-degrading protein p21, which reduces Beclin 1 consumption (Portillo et al., 2010). (III)By binding to TNFR-associated factor, CD40 activates double-stranded RNA-dependent protein kinase (PKR) which phosphorylates eukaryotic initiation factor 2 (eIF2α) and finally stimulates autophagy in *T. gondii*-infected cells *via* several mechanisms (B’Chir et al., 2013). Exploring key molecules in these pathways and mechanisms of action will provide a basis for autophagy-targeting strategies.

### 3.2 IFN-γ restricts *T. gondii* growth through autophagy-independent effects of autophagy proteins

**Figure 2:**
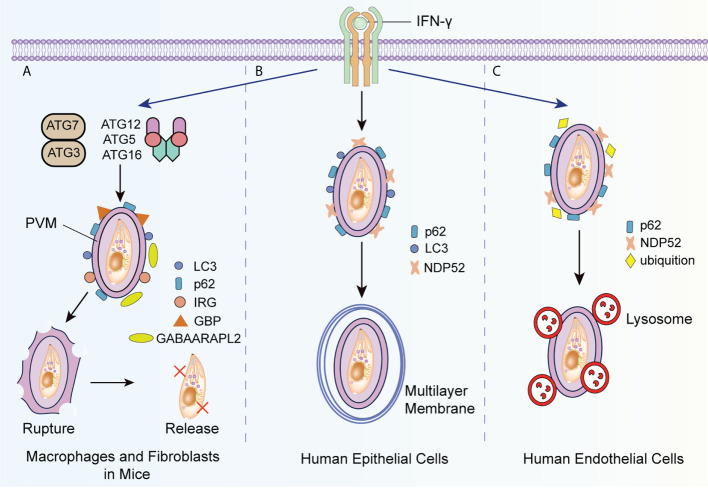
IFN-γ induced autophagic killing to T. gondii in different host cells. **(A)** IFN-γ promotes the recruitment of ATG3, ATG7 and ATG5-ATG12-ATG16L1 complex-dependent LC3, ATG8 homologs and ubiquitinated p62 to the PVM, and that of GBP and IRG to the PMV surface with the assistance of ATG8 homologs such as GABAARAPL2, causing the collapse of the PVM bubble and the release of *T. gondii* into the cytoplasm. **(B)** When type II and III *T. gondii* strains infect epithelial cells, IFN-γ recruits LC3, ubiquitinated p62, and ubiquitinated NDP52 to the PVM, and generates the multilayer membrane structure around the PVM to suppress the growth of *T. gondii*. **(C)** In human endothelial cells infected with *T. gondii*, IFN-γ promotes the recruitment of ubiquitinated p62 and NDP52 to the PVM and indues the fusion of lysosome with PV. GABAARAPL2, gamma-aminobutyric-A receptor-associated protein like 2.

IFN-γ eliminates *T. gondii* by autophagy-independent mechanisms in cells infected by different strains. IFN-γ causes PV vesiculation and rupture rather than lysosomal degradation in mouse cells infected by type II and type III *T. gondii* strains (Selleck et al., 2015). Alternatively, IFN-γ has no significant effects on cells infected by type I strains, whose specific virulence factors, (e.g. ROP5, ROP17, and ROP18), can hinder the recruitment of IRGs induced by IFN-γ (Etheridge et al., 2014). In infected mouse macrophages and fibroblasts, selective autophagy proteins, such as ATG3, ATG7, and the ATG5-ATG12-ATG16L1 complex, are necessary to recruit guanylate binding proteins (GBPs) and IRGs (Zhao et al., 2009; Khetarpal and Rader, 2014; Sasai et al., 2017). Subsequently, IRG stimulates ubiquitin deposition around the PV and results in the destruction of the PVM. IRG also recruits GBPs in a ubiquitinated protein 62 (p62)-dependent manner and promotes the killing of *T. gondii* in the cytoplasm by GBPs (**Figure 2A**) (Ling et al., 2006).

Interestingly, in human cells, IFN-γ regulates the growth and elimination of *T. gondii* by other molecular pathways. In human epithelial cells infected with type II or type III strains, IFN-γ induces the deposition of p62, nuclear dot protein 52 (NDP52), and LC3 with ATG7 and ATG16L, and forms an encapsulated PV multilayer structure to suppress the growth of *T. gondii* (**Figure 2B**) (Selleck et al., 2015). Recently, Bhushan et al. argued that this process occurs under the regulation of IFN-stimulated genes (Bhushan et al., 2020). Nevertheless, in human endothelial cells infected by type II strains, ubiquitinated K63 is recruited by IFN-γ to induce the fusion of lysosomes and PVM to *T. gondii* (Clough et al., 2016; Subauste, 2021). NDP52 and p62 are subsequently recruited around PV in the absence of LC3 and ATG16L (**Figure 2C**).

### 3.3 *T. gondii* alters autophagy targeting *via* EGFR signaling

**Figure 3:**
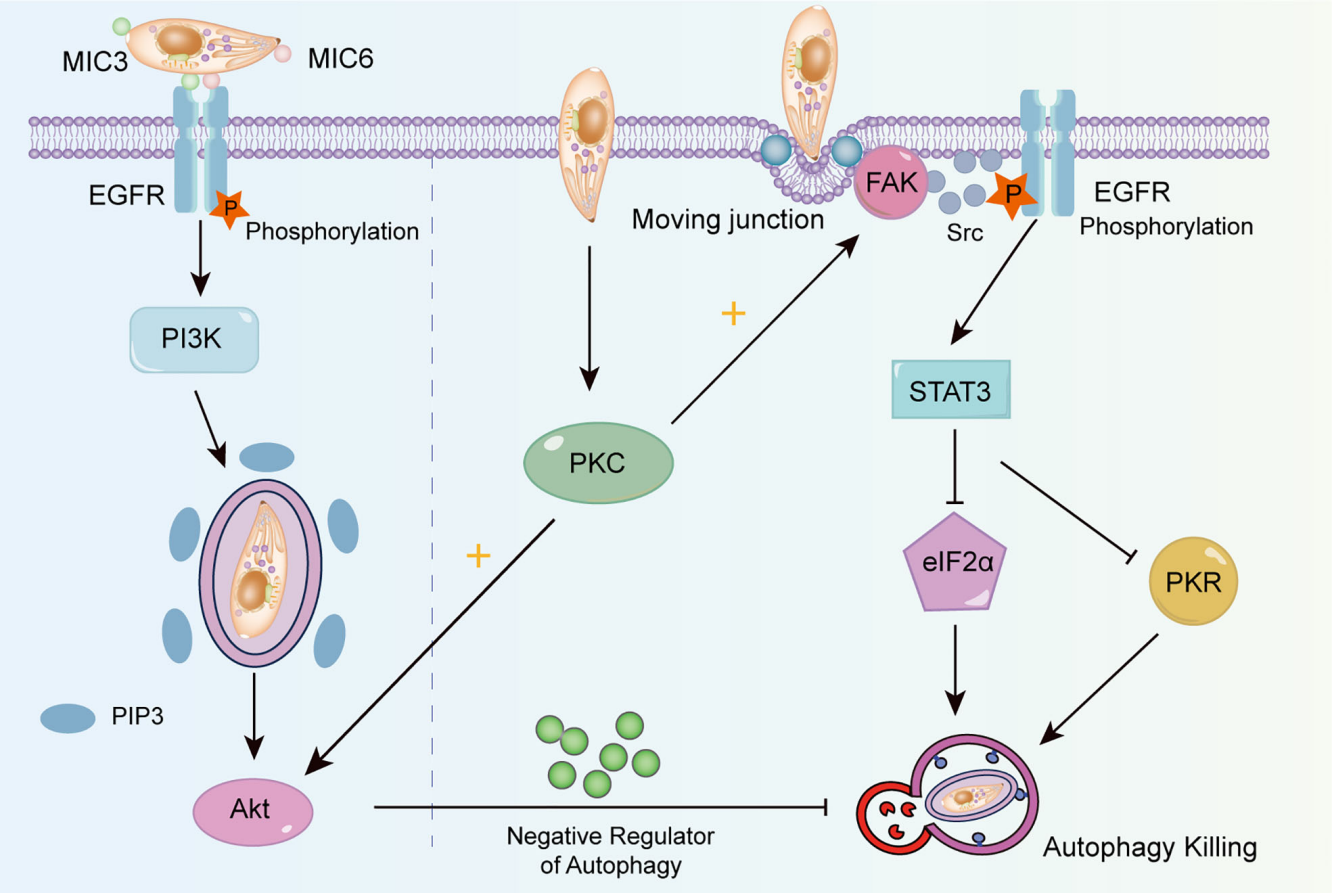
The mechanism of T. gondii changing autophagy targeting. T. gondii induces EGFR autophosphorylation through MIC3 and MIC6, triggers PI3K to produce PIP3, and then activates Akt to inhibit autophagy by producing negative regulators. The activation of FAK depends on the mobile connection formed when T. gondii invades. Activated FAK induces the transactivation of Src-dependent EGFR, and activates STAT3, thus suppressing PKR and eIF2α signaling. T. gondii can also maintain the blockade of intracellular autophagy targets by activating PKC, which helps to maintain Src signaling, EGFR autophosphorylation and Akt activation.

To avoid lysosomal degradation, *T. gondii* has developed strategies to change autophagy targeting *via* EGFR signaling. As shown in **Figure 3**, EGFR, expressed in various cells, is an important receptor to suppress cell autophagy (Purba et al., 2017). *T. gondii* leads to the phosphorylation of EGFR *via* parasitic proteins microneme (MIC) 3 and MIC6, followed by PI3K activation and PI3P accumulation (Muniz-Feliciano et al., 2013). PI3K activates the serine/threonine kinases Akt, which negatively regulates autophagy *via* the mTORC1 signaling pathway (Menon et al., 2014). Additionally, the MIC3 and MIC6 inhibit CD40-stimulated autophagy to protect parasites from autophagic degradation. Muniz-Feliciano et al. reported that parasites defective in MIC3 and MIC6 (MIC1 ko, deficient in MIC1 and secretion of MIC6; MIC3 ko, deficient in MIC3; and MIC1-3 ko, deficient in MIC1, MIC3 and secretion of MIC6) show impaired EGFR-Akt activation, indicating the dominant role of MIC3 and MIC6 in the autophagy-mediated suppression of *T. gondii* (Muniz-Feliciano et al., 2013).

Another signaling cascade activated by *T. gondii* can be utilized to avoid autophagic degradation depending on the focal adhesion kinase (FAK)/Src signal (**Figure 3**). When *T. gondii* invades cells and forms the moving junctions (Biscardi et al., 1999), FAK is phosphorylated, which in turn activates Src. This process induces the transactivation of EGFR (phospho Y845) independent of MICs (Portillo et al., 2017). Lastly, signal transducer and activator of transcription 3 (STAT3) is activated and suppresses downstream pro-autophagic PKR/eIF2α signaling (Hunter, 2011; Ullmann et al., 2013). A study has revealed that *T. gondii* activates protein kinase C (PKC) α/β to maintain the long-term activation of Src-EGFR signaling, thus effectively suppressing autophagy (Lopez Corcino et al., 2019).

## 4. Promising targets in autophagy pathway for *T. gondii* infection

It is widely accepted that stimulating autophagy ameliorates *T. gondii* infection and prevents further invasion. Current autophagy-promoting strategies can be mainly categorized into three types: promoting canonical autophagy pathway, correcting autophagy targeting, and facilitating oxidative stress to induce autophagy. Key studies and relevant mechanisms related to these strategies are summarized below.

### 4.1 Promoting canonical autophagy

Canonical autophagy signaling plays a key role in controlling host cell survival during *T. gondii* infection. It is quite possible to interfere with the replication and pathogenic effects of *T. gondii* by regulating the canonical autophagy pathway (Besteiro, 2019), in which AMPK and mTORC1 are remarkable targets.

#### 4.1.1 AMPK

The anti-*T. gondii* effect of AMPK has been widely reported. Several lines of evidence indicate that the regulation of AMPK is associated with the anti-*T. gondii* property of omega-3 polyunsaturated fatty acids (ω3-PUFAs), primarily docosahexaenoic acid (DHA). As early as 2014, Williams-Bey et al. reported that DHA promotes macrophage autophagy and thus suppresses inflammasome production (Shi et al., 2012; Williams-Bey et al., 2014). Given this, Choi et al. treated murine bone marrow-derived macrophages with DHA and found that DHA effectively enhanced the colocalization of *Toxoplasma* parasitophorous vacuoles and autophagosomes, resulting in an effective antiparasitic response (Choi et al., 2019). DHA increased the phosphorylation of AMPK, CaMKKβ, and LKB1, and specific AMPK inhibitors abrogated DHA-induce autophagy (Choi et al., 2019). Similar results were found *in vitro*, as endogenous ω3-PUFAs stimulated autophagy in bone marrow-derived macrophages, and even decreased the tissue cyst counts in the brain (Choi et al., 2019). Although ω3-PUFAs exhibited excellent antiparasitic effects in mice both *in vitro* and *in vivo*, the exact effects on humans need further investigation. AMPK probably contributes to the anti-*T. gondii* effects of statins. Although statins are well known for interfering with isoprenoid synthesis (Cortez et al., 2009; Li et al., 2013), emerging studies have demonstrated that they function as autophagy stimulators by activating AMPK and upregulating lysosomal-associated membrane protein 1 (Piplani et al., 2019). However, the role of AMPK in the effect of simvastatin has not been reported in *T. gondii* infection. Determining the relation between the antiparasitic efficacy of statins and autophagy regulation may provide new insights into the anti-*T. gondii* effects of statins (Parihar et al., 2014; Martín-Navarro et al., 2015).

#### 4.1.2 mTOR/mTORC1

As a key negative regulator of the canonical autophagy pathway, the mTOR/mTORC1 interaction has been studied extensively. The mTOR inhibitors have been widely applied in the studies aimed at enhancing autophagy (Andrade et al., 2006). Andrade and colleagues revealed that autophagy is increased in mouse macrophages treated with rapamycin, a classic mTOR inhibitor, resulting in *T. gondii* elimination in mammalian cells (Andrade et al., 2006). The mTOR inhibitor rottlerin was also shown to promote autophagy in the choriocarcinoma-derived cell line BeWo, which significantly suppressed intracellular *T. gondii* proliferation under sub-toxic concentrations (Ietta et al., 2017).

### 4.2 Correcting autophagy targeting

Accumulating research has highlighted the ability of *T. gondii* to change autophagy targeting and avoid autophagic killing (Portillo et al., 2017; Lopez Corcino et al., 2019) indicating that host-directed treatment is a plausible strategy for *T. gondii* elimination. EGFR/PI3K/AKT participates in many fundamental intracellular pathways and is an important regulator in the survival of some protozoan parasites (Penas et al., 2019; Phan et al., 2020). EGFR has emerged as a critical initial molecule in altering autophagy targeting, and EGFR targeting with EGFR tyrosine kinase inhibitors (TKIs) is a widely explored strategy. For instance, gefitinib, an EGFR TKIs with antineoplastic properties, has been applied to investigate anti-*T. gondii* activity by correcting the suppression of autophagy targeting (Lopez Corcino et al., 2019). Gefitinib promotes autophagic killing of *T. gondii* by inhibiting the EGFR/PI3K/AKT pathway, and this process is Beclin1-dependent (Lopez Corcino et al., 2019). It was further shown that the antiparasitic activity of gefitinib primarily relies on direct killing, rather than the inhibition of intracellular replication. The pharmacologic efficacy of TKIs was further evaluated *in vivo*, revealing that a relatively low dose of gefitinib could relieve cerebral toxoplasmosis in mice and control the disease process, with similar results obtained for ocular toxoplasmosis (Lopez Corcino et al., 2019). In addition to regulating autophagy, PI3K/AKT signaling may exert therapeutic effects *via* the regulation of apoptosis or cellular stress to eliminate *T. gondii* (Xu et al., 2020; Yan et al., 2021).

### 4.3 Facilitating oxidative stress

**Figure 4:**
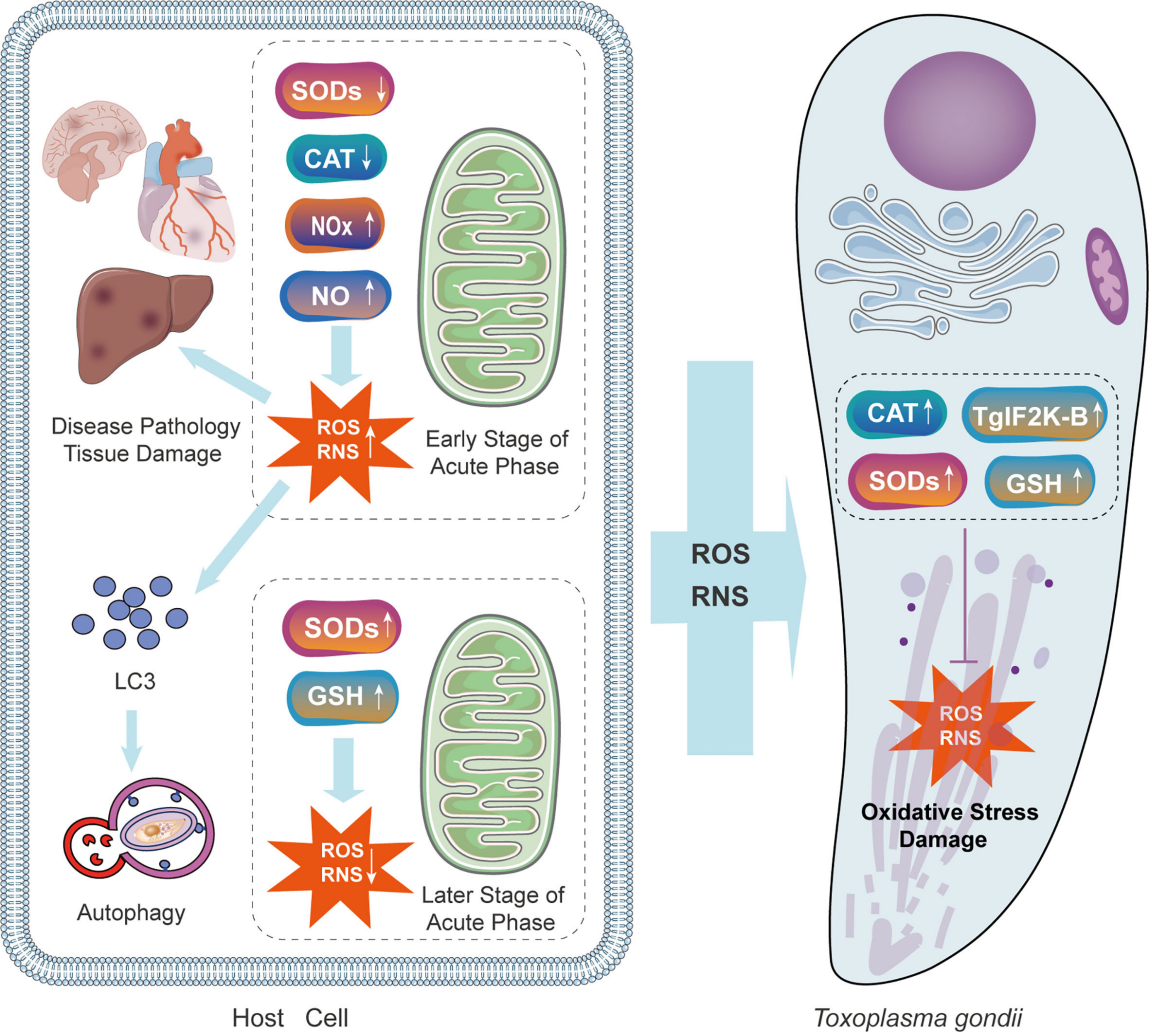
The mechanisms of oxidative injury and antioxidant defense during T. gondii infection in the parasite and the host. T. gondii infection causes immune system reaction of the host. In the early stage, the concentration of NOx and NO is raised, increasing the level of ROS and RNS. Oxidative stress caused by the host response is toxic both to parasites and the host itself. Increased ROS in host cells recruits LC3 and stimulates autophagy. In the later stage, levels of SOD and GSH are up-regulated due to the antioxidant defense of the host. Simultaneously, *T. gondii* expresses CAT, TgIF2K-B, SODs, and GSH to protect itself. GSH, glutathione; NOx, NADPH oxidase complex; TgIF2K-B, *T. gondii* eIF2α kinase.

Oxidative stress in the host cells is toxic to parasites, at the same time, it has deleterious consequences on the host (Zhuang et al., 2020). The immune response evoked by parasite infection induces the production of reactive oxygen species (ROS) which suppresses the activity of *T. gondii* in monocytes of infected animals. For instance, a high concentration of H_2_O_2_ can suppress the intracellular proliferation of tachyzoites (Szewczyk-Golec et al., 2021). In one study, resveratrol significantly limited the growth of RH tachyzoites by disrupting the redox homeostasis of parasites. Moreover, the inhibitory effects of resveratrol include releasing cellular stress, promoting apoptosis, maintaining the autophagic status of macrophages, and finally, promoting the eradication of intracellular tachyzoites by activating macrophages (Chen et al., 2019). *T. gondii* thioredoxin reductase, superoxide dismutases (SODs) (Ding et al., 2004), Catalase (CAT), and three peroxiredoxins have antioxidant functions (Akerman and Müller, 2005; Deponte and Becker, 2005; Xue et al., 2017), and enable persistent infection. ROS are intensively produced in response to the early stage of the acute phase of *T. gondii* infection, inducing oxidative stress in the tissues of the host as the defense mechanism. In the later stages of the acute phase of infection, accumulated SOD downregulate the level of ROS (**Figure 4**).

Autophagy is a catabolic process in response to different stress conditions Increasing evidence has demonstrated that oxidative stress results from a wide range of stimuli, with ROS and reactive nitrogen species (RNS) acting as the dominant intracellular signal transducers initiating autophagy (Filomeni et al., 2015). Increased ROS and RNS can recruit LC3 to autophagosomes and initiate autophagy (**Figure 4**). A certain number of studies have indicated that by facilitating oxidative stress, autophagy is a potential therapeutic target for *T. gondii*. Therefore, researchers are committed to exploring the relationship between oxidative stress and autophagic killing in *T. gondii* infection and its practical application. Methods to facilitate oxidative stress is worthy of future research. In one study myrislignan originating from nutmeg induced a redox imbalance in *T. gondii*, which led to the autophagic killing of *T. gondii* (Zhang et al., 2021a). With respect to the underlying mechanisms, myrislignan can lower the mitochondrial membrane potential as well as ATP levels in *T. gondii* and disrupt mitochondrial function (Zhang et al., 2021a). In another study, Jili-Zhang et al. found that licarin-B from nutmeg exhibits excellent anti-*T. gondii* activity by inducing mitochondrial swelling and nuclear disintegration (Zhang et al., 2021b). Consequently, licarin-B damages mitochondria and activates autophagy to kill *T. gondii* (Zhang et al., 2021b). Moreover, kijimicin decreases mitochondrial membrane potential and ROS production in *T. gondii*, thus inhibiting *T. gondii* growth (Leesombun et al., 2022). However, owing to the limited number of studies, our understanding of methods targeting oxidative stress is incomplete.

Moreover, oxidative stress caused by the host immune response exerts negative effects on the host. It is believed that antioxidant defense response of *T. gondii* helps host cells survive the attack. Consequently, antioxidants such as tryptanthrin, melatonin, and some vitamins, have been evaluated as anti-*T. gondii* therapeutics. Moon et al. demonstrated that tryptanthrin pre-treatment reduces liver cell damage by effectively decreasing ROS levels and mitochondrial dysfunction (Moon et al., 2014). Majumdar et al. reported that melatonin reduces H_2_O_2_ levels produced in the host cells *via* tert-butyl hydroperoxide. As a result, the survival of infected cells was prolonged (Majumdar et al., 2019). These studies support the beneficial effects of antioxidants for the treatment of toxoplasmosis. Further research is of great necessity to estimate the feasibility and safety of this therapy. However, research in this field is likely to have far-reaching implications for toxoplasmosis therapy.

### 4.4 Other mechanisms

In addition to the targeting mechanisms mentioned above, other promising targets with similar anti-*T. gondii* effects by affecting autophagy have been reported. Prior studies have shown that sirtuin-1 (SIRT1), a nicotine adenine dinucleotide-dependent protein deacetylase, could regulate autophagy activation (Ng and Tang, 2013). Lee and colleagues have found that the regulation of SIRT1 protein expression may exert an anti-*T. gondii* effect *via* autophagy. This phenomenon can be reversed by selective SIRT1 inhibitors (Lee et al., 2020). In another study, autophagy was enhanced by activating MutS DNA damage repair enzyme (TgMSH-1)-mediated cell cycle arrest and affecting the energy supply of the host (Lavine and Arrizabalaga, 2012). The recruitment of LC3 and ATG8 orthologs on PVM is necessary for autophagosome formation, and ATG8 labelled with a green fluorescent protein (GFP) at its amino terminus has emerged as a useful indicator of autophagy levels (Besteiro et al., 2011; Subauste, 2021). Increasing the combination of LC3-GFP and PV limits the growth of parasites by inducing the autophagic degradation of PV (Dittmar et al., 2016). Varberg et al. found that a high concentration of Plasmodium ATG3-ATG8 interaction inhibitors can promote the lipidation of ATG8, thereby stimulating autophagy in *T. gondii*-infected cells. Interestingly, inhibitors of the ATG3-ATG8 interaction exert opposite effects on *P. falciparum*-infected human hepatocytes (Hain et al., 2014; Varberg et al., 2018). Decreased incidence rate and improved clinical course of *Toxoplasma* encephalitis have been obtained by introducing protease inhibitors to the HIV treatment regimen (HIV-PIs) (Abou-El-Naga et al., 2017). Heat shock proteins (HSP), involved in the maintenance of protein homeostasis, are also potential targets for suppressing *T. gondii.* Our earlier research has shown that the downregulation of HSP29 expression levels may contribute to *T. gondii* elimination (Liu et al., 2021). Inhibiting HSP70 could significantly dysregulate host cell autophagy and decrease the reproduction of *T. gondii* (Mitra et al., 2021). Taken together, in addition to affecting common autophagy pathways, various candidate targets able to influence host cell autophagy to suppress *T. gondii* infection have been identified.

## 5. Potential drugs or chemicals promoting autophagy

Current mechanisms of promoting autophagy for anti-*T. gondii* therapy have been summarized, and we have also identified new drugs or chemicals targeting CD40 and IFN-γ to enhance autophagy with the bioinformatics approaches. Using the DrugBank database (https://go.drugbank.com/), we revealed several drugs targeting IFN-γ, and the primary agents have been summarized in **Table 1**. Surface antigen SAG1 (P30) serves as a type of major surface protein of *T. gondii* tachyzoites. SAG1 is inserted in the plasma membrane of *T. gondii* with a glycosyl-phosphatidylinositol anchor. Two glycoforms are included in the key anchor structure of the protein: Man-alpha1,2-Man-alpha1,6-Man-[GalNAc-beta1,4-]-alpha1,4-GlcN-PI and Man-alpha1,2 -Man-alpha1,6-Man [Glc-alpha1,4 -GalNAc-beta1,4-]-alpha1,4-GlcN-PI. Also, glucosamine blocks glycosyl interactions in *T. gondii* infection, thereby contributing to the acute infection of *T. gondii* tachyzoites (Zinecker et al., 2001). This was confirmed by an *in vitro* attachment assay, where soluble BSA-glucosamine blocks the attachment of SAG1 to Mabin-Darby bovine kidney cells (Robinson et al., 2004). However, the immune mechanism differs between the acute infection phase and the chronic infection phase (Robinson et al., 2004). Unfortunately, the pharmacological role of monosaccharides in *T. gondii* infection is unclear, as the nine monosaccharides making up the cell surface carbohydrates do not show clear inhibitory effects on tachyzoites infecting bovine embryonic skin and muscle cells (Crane and Dvorak, 1982). These results indicated that glucosamine might interfere with *T. gondii* infection in another form, and its mechanism of action may involve IFN-γ, which is a product of Th1/Th2 polarization in response to SAG1 antigen stimulation and facilitates the cascade immune response (Khan et al., 1988; Caetano et al., 2006; Lakhrif et al., 2018; Choi and Park, 2020).

**Table 1:** Potential Drugs targeting IFN-γ derived from the DrugBank Database.

DRUG	Generic Name	Summary	Type	Interact with IFN-γ
Glucosamine	Glucosamine	Glucosamine is a common ingredient in nutritional supplements used for the relief of joint pain.	Small Molecule	inhibitor
VIR201	VIR201	It is to facilitate the body’s immune reaction to HIV preventing progression to AIDS.	Biotech	not available
Fontolizumab	Fontolizumab	Fontolizumab (marketed under the trade name HuZAF™) is a humanized monoclonal antibody which is used as an immunosuppressive drug to treat Crohn’s disease.	Biotech	not available
Olsalazine	Olsalazine	Olsalazine is an agent for treating inflammatory bowel disease *via* inhibiting immune response.	Small Molecule	not available
Emapalumab	Emapalumab	Emapalumab is an interferon gamma blocking antibody used to treat primary hemophagocytic lymphohistiocytosis.	Biotech	neutralizer

In addition, we found that olsalazine, VIR201, and fontolizumab in the DrugBank database are predicted to interact with IFN-γ. Olsalazine increases the production of arachidonic acid metabolites *via* the cyclooxygenase pathway (i.e., prostaglandins) and lipoxygenase pathway (i.e., leukotrienes and hydroxyeicosatetraenoic acids) in patients with chronic inflammatory bowel disease, and mesalazine may reduce inflammation by blocking cyclooxygenase and inhibiting prostaglandin production in the colon. This anti-inflammatory effect of olsalazine may be related to the function of IFN-γ. VIR201 is a DNA vaccine, the second component of which is designed to increase levels of IFN-γ and improve immune function. Fontolizumab is a humanized antibody against recombinant human IFN-γ. The antibody binds to natural human IFN-γ and inhibits the expression of IFN-γ regulatory genes known to be upregulated in Crohn’s disease. Although no clear anti-*T. gondii* pharmacological effects have been reported to date, these agents should be prioritized for compound screening. High-throughput compound screening studies have shown that anti-*T. gondii* compounds with low concentrations and low levels of IFN-γ evaluated by *in vitro* parasite growth inhibition assays were likely to affect autophagy-related molecules such as LC3 (Radke et al., 2018). Therefore, focusing on autophagy-related molecules is the next step in the discovery of drugs that have synergistic effects with IFN-γ.

We were unable to identify drugs targeting CD40; however, many compounds inhibit both IFN-γ and CD40 and are potential anti-*T. gondii* drugs (see **Table S1** for a summary of specific interactions of each agent). These potential compounds that interfere with CD40 could serve as molecular manipulation tools to clarify the role of CD40 in the autophagy of *T. gondii*-infected cells and to identify compounds with therapeutic effects. Of note, simvastatin is among the compounds that may affect both IFN-γ and CD40. Although some reports have indicated that simvastatin inhibits *T. gondii* adherence, invasion, and proliferation in HeLa cells (Sanfelice et al., 2017; Sanfelice et al., 2019), the pharmacological mechanism of action has not been determined. Paclitaxel (dihydroquercetin) and a dihydrofolate inhibitor (ethacrynic acid) suppress the growth of rapidly proliferating *T. gondii* RH strains *in vitro* (Abugri et al., 2018). Curcumin is also an active ingredient in Chinese herbal medicine that inhibits the growth of tachyzoites *in vitro* (Saki et al., 2020). It could also reduce DNA methylation induced by *T. gondii* (Saki et al., 2020), and is a potentially effective agent for chronic *T. gondii* infection (Azami et al., 2018; El-Shafey et al., 2020). These compounds have varying degrees of efficacy against *T. gondii*; however, the specific mechanisms of action are not clearly defined, hindering their clinical application. Since IFN-γ and CD40 are central components in the autophagy-related mechanism underlying anti-*T. gondii* infection, future studies should evaluate the anti-*T. gondii* pharmacological effects of these plant-derived compounds targeting IFN-γ and CD40.

## 6. Challenges and opportunities

Autophagy singling exerts complex interactions with *T. gondii* survival, and accumulating studies have focused on autophagy as a target to eliminate *T. gondii*. However, established strategies are generally confined to activating autophagy *via* chemical substances, without sufficient consideration of *T. gondii*-mediated autophagy suppression. Thus, limited and unstable effects have been obtained.

Also, future autophagy-promoting anti-*T. gondii* drug research should consider strategies that incorporate highly specific key proteins. For example, AR12, which induces autophagy and reduces EGFR expression in SARS-COV-2-infected Vero cells, may have similar effects on *T. gondii* infection (Rayner et al., 2020). Reported drugs do not cover all inhibitors of potential targets, and ATG4, as the only protease of the core mammalian autophagy proteins, is closely associated with cancer, neurodegeneration, and microbial infection. Various *in vivo* and *in vitro* methods to detect ATG4 activity have been established in the last decade (Li et al., 2011; Fu et al., 2019). Moreover, ATGB is a potent target for suppressing *T. gondii*, as evidenced by the antiproliferative effects of an ATG4B agonist in MDA-MB-231 cells (Pengo et al., 2018). Anti-*T. gondii* therapies targeting ATG4 are promising and should be a focus of basic research and clinical trials.

Since *T. gondii* has a long history of coexistence and antagonistic relationships with human hosts, therapies targeting autophagy should consider the evolution of *T. gondii* Strategies aimed at the complete removal of *T. gondii* intracellularly or *in vivo* for a certain period using methods independent of autophagy modulation can lead to drug resistance and the development of super-virulent *T. gondii*. To avoid this problem, monitoring genetic changes in *T. gondii* and the use of multi-targeted agents are important strategies. Additionally, a shift in emphasis from killing *T. gondii* to the removal of *T. gondii* pathogenicity may provide a basis for the next generation of anti-*T. gondii* therapies. To this end, further elucidation of the mechanisms underlying *T. gondii* pathogenesis will be the key to the development of novel treatment strategies.

## 7. Conclusion

Given the important antiparasitic effects of autophagy, it is not surprising that modulation of autophagy serves as a therapeutic strategy for *T. gondii* infection. Autophagy-related molecules, including CD40, IFN-γ, EGFR, mTORC1, and AMPK, as well as oxidative stress are potential targets. A series of drugs and chemicals have been developed to promote autophagy against *T. gondii* infection, with clearly demonstrated pharmacological value. However, these strategies are still in their early stages, and more precise targets and better assessments of pharmacological effects are needed. In addition, the possibility of drug resistance should be evaluated in future studies.

## Author contributions

AC: Conceptualization, Writing—original draft preparation, review and editing, Project administration. HZ, BC, SZ, HW, YS, and SY contributed to the paper writing and revisions. LJ and ML: Conceptualization, Writing—review and editing, Project administration, Funding acquisition. All authors have read and agreed to the published version of the manuscript.

## Funding

This work was supported by the National Natural Science Foundation of China (grant number 32170510), the Natural Science Foundation of Hunan Province, China (grant number 2020JJ4765), the Open Sharing Fund for the Large-scale Instruments and Equipment of Central South University (grant number CSUZC202236), Science and Technology Program of Hunan Province (project number 2021ZK4154), Graduate Case base construction project of Central South University (project number 2020ALK91), and Innovation Training Program of Central South University (project number 20220026020009).

## Acknowledgments

We would like to thank Editage (www.editage.cn) for English language editing.

## Conflict of interest

The authors declare that the research was conducted in the absence of any commercial or financial relationships that could be construed as a potential conflict of interest.

## Publisher’s note

All claims expressed in this article are solely those of the authors and do not necessarily represent those of their affiliated organizations, or those of the publisher, the editors and the reviewers. Any product that may be evaluated in this article, or claim that may be made by its manufacturer, is not guaranteed or endorsed by the publisher.
