# Supplementary material for: Modulation of autophagy as a therapeutic strategy for Toxoplasma gondii infection
Source: Front Cell Infect Microbiol. 2022 Aug 24;12:902428. doi: 10.3389/fcimb.2022.902428 (PMC9448867; doi:10.3389/fcimb.2022.902428)
Supplement: Supplementary file 1 [file Table_1.docx]

Table S1. Chemicals interacting with IFN-γ and CD40

| Drugs | Interact with IFN-γ | Interact with CD40 |
| --- | --- | --- |
| Simvastatin | Yes | Yes |
| Mercury | Yes | Yes |
| Histamine | Yes | Yes |
| Acetylmuramyl-Alanyl-Isoglutamine | Yes | Yes |
| Poly I-C | Yes | Yes |
| Antirheumatic Agents | Yes | Yes |
| Vincristine | Yes | Yes |
| Arsenic | Yes | Yes |
| lipopolysaccharide, E. coli O26-B6 | Yes | Yes |
| Asbestos | Yes | Yes |
| nickel sulfate | Yes | Yes |
| Betamethasone Valerate | Yes | Yes |
| Quercetin | Yes | Yes |
| Calcimycin | Yes | Yes |
| Thimerosal | Yes | Yes |
| Calcitriol | Yes | Yes |
| zoledronic acid | Yes | Yes |
| Curcumin | Yes | Yes |
| lipopolysaccharide, E coli O55-B5 | Yes | Yes |
| decitabine | Yes | Yes |
| Lipopolysaccharides | Yes | Yes |
| Demecolcine | Yes | Yes |
| Nickel | Yes | Yes |
| Dronabinol | Yes | Yes |
| Particulate Matter | Yes | Yes |
| Drugs, Chinese Herbal | Yes | Yes |
| Polysaccharides | Yes | Yes |
| entinostat | Yes | Yes |
| Ribomunyl | Yes | Yes |
| FK 565 | Yes | Yes |
| Tetradecanoylphorbol Acetate | Yes | Yes |
| Fluorouracil | Yes | Yes |
| Tretinoin | Yes | Yes |
| Formaldehyde | Yes | Yes |
| gemcitabine | Yes | Yes |
| Zinc | Yes | Yes |
| 1,2-bis(2-aminophenoxy)ethane N,N,N',N'-tetraacetic acid acetoxymethyl ester | Yes | No |
| 15-deoxy-delta(12,14)-prostaglandin J2 | Yes | No |
| 15-deoxyprostaglandin J2 | Yes | No |
| 2-(2-amino-3-methoxyphenyl)-4H-1-benzopyran-4-one | Yes | No |
| 22-hydroxycholesterol | Yes | No |
| 2,4,5,2',4',5'-hexachlorobiphenyl | Yes | No |
| 2-(4-morpholinyl)-8-phenyl-4H-1-benzopyran-4-one | Yes | No |
| 2-amino-1-methyl-6-phenylimidazo(4,5-b)pyridine | Yes | No |
| 2-amino-5,6-dihydro-6-methyl-4H-1,3-thiazine | Yes | No |
| 2-aminoethoxydiphenyl borate | Yes | No |
| 2-butenal | Yes | No |
| 2-hydroxy-4-(2,2,3,3,3-pentafluoropropoxy)benzoic acid | Yes | No |
| 2-methyl-2H-pyrazole-3-carboxylic acid (2-methyl-4-o-tolylazophenyl)amide | Yes | No |
| 2-morpholin-4-yl-6-thianthren-1-yl-pyran-4-one | Yes | No |
| 3,3',4,5'-tetrahydroxystilbene | Yes | No |
| 3-(3-chloro-4-methoxyphenyl)-1,1-dimethylurea | Yes | No |
| 3-(4-methylphenylsulfonyl)-2-propenenitrile | Yes | No |
| 3,5-bis(2-fluorobenzylidene)piperidin-4-one | Yes | No |
| 3-chlorophenol | Yes | No |
| 3-Iodobenzylguanidine | Yes | No |
| 4-(4-fluorophenyl)-2-(4-hydroxyphenyl)-5-(4-pyridyl)imidazole | Yes | No |
| 4-bromophenacyl bromide | Yes | No |
| 4-methylhistamine | Yes | No |
| 4-nitrososulfamethoxazole | Yes | No |
| 4-nonylphenol | Yes | No |
| abacavir | Yes | No |
| Acetylcysteine | Yes | No |
| Acrolein | Yes | No |
| acteoside | Yes | No |
| Active Hexose Correlated Compound | Yes | No |
| acyline | Yes | No |
| Adenine | Yes | No |
| Air Pollutants | Yes | No |
| Air Pollutants, Occupational | Yes | No |
| alclometasone dipropionate | Yes | No |
| alitretinoin | Yes | No |
| allergovit | Yes | No |
| alpha-cyano-(3,4-dihydroxy)-N-benzylcinnamide | Yes | No |
| alpha-methylhistamine | Yes | No |
| Amb a I protein, Ambrosia artemisiifolia | Yes | No |
| Amiloride | Yes | No |
| ammonium trichloro(dioxoethylene-O,O'-)tellurate | Yes | No |
| Amphotericin B | Yes | No |
| anandamide | Yes | No |
| anthra(1,9-cd)pyrazol-6(2H)-one | Yes | No |
| anthranilic acid | Yes | No |
| Antigens, Dermatophagoides | Yes | No |
| Antigens | Yes | No |
| Anti-HIV Agents | Yes | No |
| Antimony Sodium Gluconate | Yes | No |
| arsenic trioxide | Yes | No |
| arsenite | Yes | No |
| Aspirin | Yes | No |
| Atrazine | Yes | No |
| Azacitidine | Yes | No |
| BCH 1393 | Yes | No |
| Benzene | Yes | No |
| Benzo(a)pyrene | Yes | No |
| beryllium sulfate | Yes | No |
| beta-Glucans | Yes | No |
| bioallethrin | Yes | No |
| bismuth tripotassium dicitrate | Yes | No |
| bisphenol A | Yes | No |
| Budesonide | Yes | No |
| Butyrates | Yes | No |
| cadmium acetate | Yes | No |
| Cadmium Chloride | Yes | No |
| Cadmium | Yes | No |
| caffeic acid phenethyl ester | Yes | No |
| Calcium | Yes | No |
| Cannabidiol | Yes | No |
| Carbamates | Yes | No |
| carvedilol | Yes | No |
| Cholecalciferol | Yes | No |
| chromium hexavalent ion | Yes | No |
| Cilomilast | Yes | No |
| Cimetidine | Yes | No |
| Ciprofloxacin | Yes | No |
| Cisplatin | Yes | No |
| Citrinin | Yes | No |
| Clarithromycin | Yes | No |
| clobenpropit | Yes | No |
| Clofibrate | Yes | No |
| Clozapine | Yes | No |
| Cobalt | Yes | No |
| Cocaine | Yes | No |
| Colforsin | Yes | No |
| Cycloheximide | Yes | No |
| Cyclosporine | Yes | No |
| Cyclosporins | Yes | No |
| cyfluthrin | Yes | No |
| Cytochalasin D | Yes | No |
| Dactinomycin | Yes | No |
| Dasatinib | Yes | No |
| delta-8-tetrahydrocannabinol | Yes | No |
| deoxynivalenol | Yes | No |
| Dexamethasone | Yes | No |
| Dichloroacetic Acid | Yes | No |
| Diclofenac | Yes | No |
| Dimaprit | Yes | No |
| Dimethyl Fumarate | Yes | No |
| Dimethyl Sulfoxide | Yes | No |
| Dinoprostone | Yes | No |
| Diuron | Yes | No |
| Docosahexaenoic Acids | Yes | No |
| doxifluridine | Yes | No |
| Dust | Yes | No |
| Dydrogesterone | Yes | No |
| emulphogene BC 720 | Yes | No |
| enterotoxin A, Staphylococcal | Yes | No |
| enterotoxin B, staphylococcal | Yes | No |
| enterotoxin F, Staphylococcal | Yes | No |
| Environmental Pollutants | Yes | No |
| Eosine I Bluish | Yes | No |
| epinastine | Yes | No |
| Estradiol | Yes | No |
| Estrogens, Conjugated (USP) | Yes | No |
| Ethylmercury Compounds | Yes | No |
| Ethyl Methanesulfonate | Yes | No |
| Famotidine | Yes | No |
| FEC protocol | Yes | No |
| Fenitrothion | Yes | No |
| fluoranthene | Yes | No |
| Flurbiprofen | Yes | No |
| Fluticasone | Yes | No |
| gadodiamide | Yes | No |
| gadolinium chloride | Yes | No |
| Gadolinium DTPA | Yes | No |
| Gemfibrozil | Yes | No |
| Genistein | Yes | No |
| ginsenoside Re | Yes | No |
| Gliotoxin | Yes | No |
| Glucosamine | Yes | No |
| Glutathione | Yes | No |
| GW0072 | Yes | No |
| GW280264X | Yes | No |
| GW 7647 | Yes | No |
| Hemagglutinins | Yes | No |
| Heme | Yes | No |
| Hemin | Yes | No |
| herbimycin | Yes | No |
| Heroin | Yes | No |
| hesperetin | Yes | No |
| Hexachlorobenzene | Yes | No |
| Hydrocortisone | Yes | No |
| Hydrogen Peroxide | Yes | No |
| Ibuprofen | Yes | No |
| Imatinib Mesylate | Yes | No |
| indirubin | Yes | No |
| Indomethacin | Yes | No |
| indomethacin morpholinylamide | Yes | No |
| Ionomycin | Yes | No |
| irbesartan | Yes | No |
| Iron Chelating Agents | Yes | No |
| Iron | Yes | No |
| Isocyanates | Yes | No |
| isoliquiritigenin | Yes | No |
| isopentenyl pyrophosphate | Yes | No |
| Isoproterenol | Yes | No |
| jasplakinolide | Yes | No |
| JHW 015 | Yes | No |
| lactacystin | Yes | No |
| Lamivudine | Yes | No |
| Lansoprazole | Yes | No |
| Latex | Yes | No |
| latrunculin B | Yes | No |
| lead acetate | Yes | No |
| lead chloride | Yes | No |
| licochalcone A | Yes | No |
| Lindane | Yes | No |
| Linoleic Acid | Yes | No |
| Lipid A | Yes | No |
| lipopolysaccharide, Escherichia coli O111 B4 | Yes | No |
| Lisuride | Yes | No |
| lupane | Yes | No |
| mecoprop | Yes | No |
| Medroxyprogesterone Acetate | Yes | No |
| Mercuric Chloride | Yes | No |
| Methanol | Yes | No |
| Methotrexate | Yes | No |
| methyl isocyanate | Yes | No |
| Methylmercury Compounds | Yes | No |
| Mevalonic Acid | Yes | No |
| Mifepristone | Yes | No |
| monocyte locomotion inhibitory factor | Yes | No |
| monomethylpropion | Yes | No |
| morin | Yes | No |
| moxifloxacin | Yes | No |
| N-((2-(hydroxyaminocarbonyl)methyl)-4-methylpentanoyl)-3-(2'-naphthyl)alanylalanine, 2-aminoethylamide | Yes | No |
| N-3-(aminomethyl)benzyl)acetamidine | Yes | No |
| N-(6-methylamino-3-nitrophenyl)-3-(3-indolyl)acrylamide | Yes | No |
| naringin | Yes | No |
| nefazodone | Yes | No |
| Nevirapine | Yes | No |
| Niacin | Yes | No |
| nickel chloride | Yes | No |
| nimesulide | Yes | No |
| Nitric Oxide Donors | Yes | No |
| Nitric Oxide | Yes | No |
| Nitrites | Yes | No |
| Nitroprusside | Yes | No |
| nivalenol | Yes | No |
| N, N, N', N'-tetrakis (2-pyridylmethyl)ethylenediamine | Yes | No |
| NVP ABE171 | Yes | No |
| oleanolic acid 3-acetate | Yes | No |
| Omeprazole | Yes | No |
| orazipone | Yes | No |
| Organometallic Compounds | Yes | No |
| Organothiophosphates | Yes | No |
| Oxygen | Yes | No |
| Ozone | Yes | No |
| Pam(3)CSK(4) peptide | Yes | No |
| Patulin | Yes | No |
| PD 0325901 | Yes | No |
| perfluorooctane sulfonic acid | Yes | No |
| Perfume | Yes | No |
| Phenylbutyrates | Yes | No |
| Phloretin | Yes | No |
| Picibanil | Yes | No |
| pimecrolimus | Yes | No |
| pioglitazone | Yes | No |
| Piperonyl Butoxide | Yes | No |
| Plant Extracts | Yes | No |
| Plant Preparations | Yes | No |
| Plicamycin | Yes | No |
| Polycyclic Hydrocarbons, Aromatic | Yes | No |
| polydatin | Yes | No |
| poractant alfa | Yes | No |
| Prednisone | Yes | No |
| Progesterone | Yes | No |
| pterostilbene | Yes | No |
| pyrene | Yes | No |
| pyrrolidine dithiocarbamic acid | Yes | No |
| ranitidine bismuth citrate | Yes | No |
| Ranitidine | Yes | No |
| Reactive Oxygen Species | Yes | No |
| resiquimod | Yes | No |
| resveratrol | Yes | No |
| Ribavirin | Yes | No |
| ribosome inactivating protein, Viscum | Yes | No |
| rimexolone | Yes | No |
| Ritonavir | Yes | No |
| Ro 41-5253 | Yes | No |
| rosiglitazone | Yes | No |
| Rotenone | Yes | No |
| Rutin | Yes | No |
| SB 203580 | Yes | No |
| Silver | Yes | No |
| Silymarin | Yes | No |
| Simazine | Yes | No |
| Sirolimus | Yes | No |
| S-Nitrosoglutathione | Yes | No |
| sodium arsenite | Yes | No |
| Sodium Azide | Yes | No |
| sodium bichromate | Yes | No |
| Soot | Yes | No |
| Spironolactone | Yes | No |
| SR 144528 | Yes | No |
| Sulfasalazine | Yes | No |
| Tacrolimus | Yes | No |
| taxifolin | Yes | No |
| Terbutaline | Yes | No |
| Tetanus Toxoid | Yes | No |
| Tetrachloroethylene | Yes | No |
| Thalidomide | Yes | No |
| Theophylline | Yes | No |
| thymoquinone | Yes | No |
| tin protoporphyrin IX | Yes | No |
| titanium dioxide | Yes | No |
| Titanium | Yes | No |
| TO-901317 | Yes | No |
| Tobacco Smoke Pollution | Yes | No |
| tolmetin glucuronide | Yes | No |
| toxB protein, Clostridium difficile | Yes | No |
| Trichloroacetic Acid | Yes | No |
| Trichloroethylene | Yes | No |
| trichostatin A | Yes | No |
| tripterine | Yes | No |
| troglitazone | Yes | No |
| trovafloxacin | Yes | No |
| U 0126 | Yes | No |
| Urethane | Yes | No |
| Ursodeoxycholic Acid | Yes | No |
| Vaccines, Virus-Like Particle | Yes | No |
| Vehicle Emissions | Yes | No |
| wortmannin | Yes | No |
| Y 27632 | Yes | No |
| zafirlukast | Yes | No |
| ZD 9331 | Yes | No |
| Zidovudine | Yes | No |
| Zinc Sulfate | Yes | No |
| ZM 241385 | Yes | No |
| zomepirac glucuronide | Yes | No |
| Zymosan | Yes | No |
| 1-(6-((3-methoxyestra-1,3,5(10)-trien-17-yl)amino)hexyl)-1H-pyrrole-2,5-dione | No | Yes |
| ((1S,2S,4R)-4-(4-((1S)-2,3-dihydro-1H-inden-1-ylamino)-7H-pyrrolo(2,3-d)pyrimidin-7-yl)-2-hydroxycyclopentyl)methyl sulphamate | No | Yes |
| 2,3-bis(palmitoyloxy)-2-propyl-N-palmitoyl-cysteinyl-seryl-seryl-asparaginyl-alanine | No | Yes |
| 4-(5-benzo(1,3)dioxol-5-yl-4-pyridin-2-yl-1H-imidazol-2-yl)benzamide | No | Yes |
| 4-diphenylacetoxy-1,1-dimethylpiperidinium | No | Yes |
| (6-(4-(2-piperidin-1-ylethoxy)phenyl))-3-pyridin-4-ylpyrazolo(1,5-a)pyrimidine | No | Yes |
| Adjuvants, Immunologic | No | Yes |
| Aldehydes | No | Yes |
| alpha-fluoromethylhistamine | No | Yes |
| Aluminum Hydroxide | No | Yes |
| Apigenin | No | Yes |
| Arachidonic Acid | No | Yes |
| Ascorbic Acid | No | Yes |
| Atorvastatin Calcium | No | Yes |
| Atropine | No | Yes |
| BAY 11-7085 | No | Yes |
| bicalutamide | No | Yes |
| butyraldehyde | No | Yes |
| Carbachol | No | Yes |
| Dihydrotestosterone | No | Yes |
| Dinitrochlorobenzene | No | Yes |
| Eicosapentaenoic Acid | No | Yes |
| Fatty Acids, Unsaturated | No | Yes |
| Fenofibrate | No | Yes |
| fisetin | No | Yes |
| Fluorides | No | Yes |
| Gold Sodium Thiosulfate | No | Yes |
| helenalin | No | Yes |
| (+)-JQ1 compound | No | Yes |
| Luteolin | No | Yes |
| LW 50020 | No | Yes |
| mercuric bromide | No | Yes |
| Paraquat | No | Yes |
| pentanal | No | Yes |
| phenethyl isothiocyanate | No | Yes |
| Phenylmercuric Acetate | No | Yes |
| Pirenzepine | No | Yes |
| PLX4032 | No | Yes |
| propionaldehyde | No | Yes |
| Silicon Dioxide | No | Yes |
| Sodium Dodecyl Sulfate | No | Yes |
| tesmilifene | No | Yes |
| tetrathiomolybdate | No | Yes |
| tiotidine | No | Yes |
| titanium nickelide | No | Yes |
| Triprolidine | No | Yes |
| Valproic Acid | No | Yes |
| vorinostat | No | Yes |
